# Biodiversity policy and subnational implementation in the remote regions of France

**DOI:** 10.1007/s10668-022-02502-4

**Published:** 2022-06-30

**Authors:** Gianluca Ferraro, Pierre Failler, Gregoire Touron-Gardic

**Affiliations:** University of Portsmouth, Centre for Blue Governance, Richmond Building, Portland Street, Portsmouth, PO1 3DE UK

**Keywords:** Biodiversity, Policy implementation, Multilayer governance, Regions, Europe

## Abstract

Biological diversity in the marine and coastal environment is declining globally. Several layers of governance intertwine in the regulation of biodiversity with multiple strains of policy developments taking place at the international, national and subnational levels. In particular, the subnational level of governance has become crucial in the implementation of biodiversity protection. The article aims at better understanding how governance mechanisms in defence of biodiversity can be developed and implemented at the subnational level and what obstacles they may face. For this purpose, the article relies on a single-case study: it analyses biodiversity policy in France and explains its subnational implementation in Reunion. Major achievements and impediments are also discussed for policy tools adopted for the protection of areas and species. The study reveals important bureaucratic, political and societal pressures that can affect subnational implementation together with the availability of material and immaterial resources. The article concludes with policy recommendations that are specific to Reunion but concern aspects common to other Outermost Regions of the European Union: centre-local coordination, regional strategy, public engagement and transnational collaboration.

## Introduction

Oceans and their ecosystems provide multiple services to the humankind. They offer food, regulate the climate, provide raw materials and can function as a source of renewable energy and a way for transportation. They also play an important role in recreational activities and cultural identity (EIU, 2015; EU, 2017). Unfortunately, biological diversity in the marine and coastal environment is declining globally due to human activities and despite the efforts in terms of public policy put in place to address this problem (IUCN, 2018). Several layers of governance intervene and intertwine in the regulation of biodiversity with multiple strains of policy developments taking place at the international, national and subnational levels.

At the international level, global governance arrangements regulate, directly or indirectly, biodiversity protection and are complemented by regional institutions and organisations. In Europe, a complex system of legal acts and political documents (in the form of directives, policies, communications and programmes) has been adopted by the European Union (EU) to address biodiversity loss on land and in the seas of its 27 Member States (Rouillard et al., 2016). However, both global and regional institutions can be effective only if national governments comply by adopting and applying legislations and public policies against environmental degradation in areas following under their national jurisdiction (Hagerman & Pelai, 2016; Young, 1999). In this process of domestic implementation, subnational governments play a crucial role as acknowledged by the Conference of the Parties of the Convention on Biological Diversity (CBD) (Puppim de Oliveira et al., 2011).

The implementation of international and national biodiversity policies at the subnational level of governance is particularly relevant in the Outermost Regions^1^ (ORs) of the EU. The remoteness and insularity of these regions have made them biodiversity hotspots for the variety of flora and fauna they host (Benzaken & Renard, 2011). Nevertheless, the geographical isolation and small size of the ORs also limit their policy action (e.g. because of limited technical capacity) (Azevedo, 2017).

**Fig. 1:**
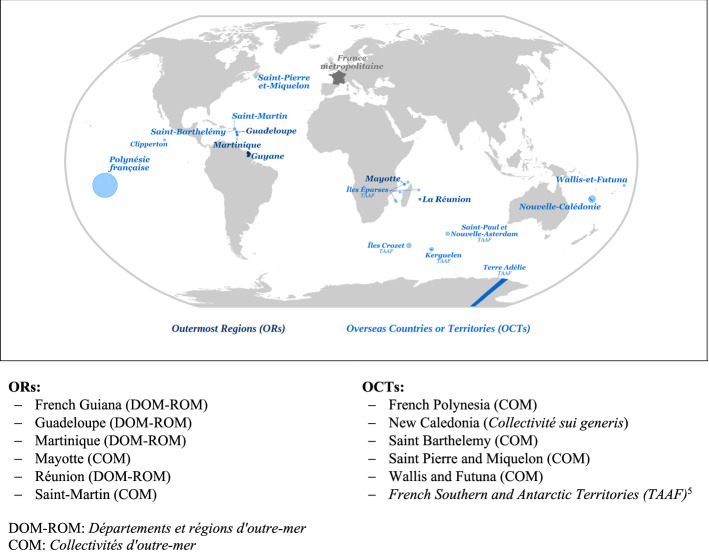
France’s overseas entities (Based on *Loi constitutionnelle n°2003–276 du 28 mars 2003 relative à l'organisation décentralisée de la République*. See also https://www.senat.fr/role/fiche/cl_outre_mer.html (last access: 17.09.2021))

Within the EU, France is the European country with the largest surface (i.e. 549,000 km^2^). The country has also more than 10 million km^2^ of maritime area under its jurisdiction, which makes it the second-largest maritime state of the world after the USA (OECD, 2016b). Thanks to this terrestrial and maritime extension and its geographical position across all oceans, France hosts a large variety of terrestrial and marine ecosystems (MEDDTL, 2011). In particular, French overseas entities are home to a very rich biodiversity (Benzaken & Renard, 2011). These overseas entities include ORs and overseas countries and territories (OCTs) of the EU; they are mainly islands^2^ located in the Atlantic, Pacific and Indian oceans (Fig. 1). While altogether these overseas areas of France represent one quarter of the French metropolitan territory, only a small percentage of the French population (about 4%) lives in these areas in 2022.^3^

France also figures among the ten countries with the greatest numbers of habitats in unfavourable state and endangered species. Pressures on the environment (e.g. habitats degradation and pollution) coming from intensive agriculture, urbanisation, transport infrastructures and overfishing are particularly intense in overseas France where they are threatening rich biodiversity hotspots (OECD 2016a; b). Biodiversity in overseas France is particularly vulnerable because most of these territories are islands exposed to sea-level rise due to climate change (MEDDTL, 2011; Nazarnia et al., 2020).

For its geographical extension, relevance for biodiversity and state of its environment, France has significant responsibilities in the area of nature conservation on a global scale (MEDDTL, 2011), which makes it a relevant case for the study of biodiversity policy implementation. In particular, its ORs deserve more scholarly attention. Although crucial for the global environment with their rich biodiversity, they are often neglected by the academic literature on biodiversity policy. Only very few reports have been produced by international governmental and non-governmental organisations (e.g. EU and IUCN, respectively). This article attempts to cover such gap in the literature. It focuses on one of the French ORs, i.e. Reunion, for three reasons. First, it is located in one of the top ten world marine biodiversity hotspots (IUCN, 2018). Second, this OR has shown more policy dynamism compared to other French overseas entities. Third, Reunion is part of the Indian Ocean Commission and has promoted regional cooperation for marine environmental research and the Blue Economy within this ocean basin (Failler, 2020).

The article makes a novel contribution to the scientific literature since it is the first in-depth analysis of biodiversity policy implementation in Reunion. With the exception of Lagabrielle’s (2007) work on the planning of biodiversity conservation in Reunion, research with this focus and geographical scope is scarce if non-existent. Some policy reports have been produced, for instance, in the framework of the BEST Initiative and IFRECOR^4^ (Trégarot & Failler, 2021; Trégarot et al., 2015), while a few articles discuss the policy responses to climate change (Magnan & Duvat, 2018; Ribalaygua et al., 2019). However, most of this research precedes important policy changes that have occurred in France and Reunion in the last few years. While focusing on Reunion, the article also discusses the influence of mainland France on the delivery of policies in its overseas entities, particularly the ORs of the EU. At a higher level of abstraction, it attempts to determine the factors hindering implementation at the subnational level of governance and recommend how such obstacles can be overcome. The purpose of the article is, ultimately, to serve both the theory and the practice of public policy. Indeed, the whole object of policy research is ‘to arrive at some policy recommendations’ (Bardach, 2012: 95).

After framing theoretically the issue of subnational implementation, the article clarifies the methodology adopted, explores biodiversity policy in France and explains its implementation in a multi-layered context. The case of Reunion is analysed in detail with the purpose of identifying major constraints in subnational implementation. Finally, the article provides a set of policy recommendations that are specific to Reunion but can claim for an effort of generalisation to other ORs of France and the EU.

## Policy implementation and the subnational level of governance

In the absence of a general theory of policy implementation, the topic has been studied in policy research from several analytical approaches since it appeared in the academic literature in the 1970s (O’Toole, 2000). A first approach has looked at policy implementation from a hierarchical perspective that uses leadership and control to explain implementation as the simple administrative execution (at the bottom) of political decisions (taken at the top of the central government) (Younis & Davidson, 1990). As a response to this top-down perspective, a bottom-up approach to the study of policy implementation has paid more attention to the lower levels of bureaucracy such as the local implementors and street-level bureaucrats directly involved in the delivery of a policy and the services it entails (Lipsky, 1980; Parsons, 1995). This same approach has also recognised the relevance of the target groups of a policy initiative (Hjern & Porter, 1981). For these reasons, scholars within the bottom-up tradition have given greater attention to negotiation between policy-makers and administrative implementing agencies, and participation of all actors targeted by a public programme (Howlett & Ramesh, 2003). Despite their divide, the contributions coming from these two perspectives revealed that policy implementation is a complex process that stands between “central guidance” and “local autonomy” (Pülzl & Treib, 2006). This acknowledgement called for new analytical frameworks that could study policy implementation by combining insight from both approaches (e.g. Goggin et al., 1990; Matland, 1995; Winter, 1990).

In fact, the role of actors and organisations located at lower territorial levels in the administrative structure of a country has been stressed as an important part of the implementation process since the early days of the implementation research. In general, more actors at the subnational level (potentially acting as veto points) can impact negatively on implementation and the achievement of policy objectives due to the “complexity of joint action” (Pressman & Wildavsky, 1973). It follows that the response of subnational agencies to central policy mandates and the coordination among them is a pre-requisite for avoiding implementation failures (Winter, 1990). Indeed, policy directions in the form of national laws and strategies are decided by central governments and reach subnational governments. Here, they face contextual politics (played by a complex set of both public and private actors) and the capacity of the subnational territorial unit empowered with a definite amount of resources that are both physical (e.g. personnel) and immaterial (like knowledge and skills) (Goggin et al., 1990). Therefore, *subnational politics* and *subnational capacity* influence policy implementation and determine its (policy) results.

**Fig. 2:**
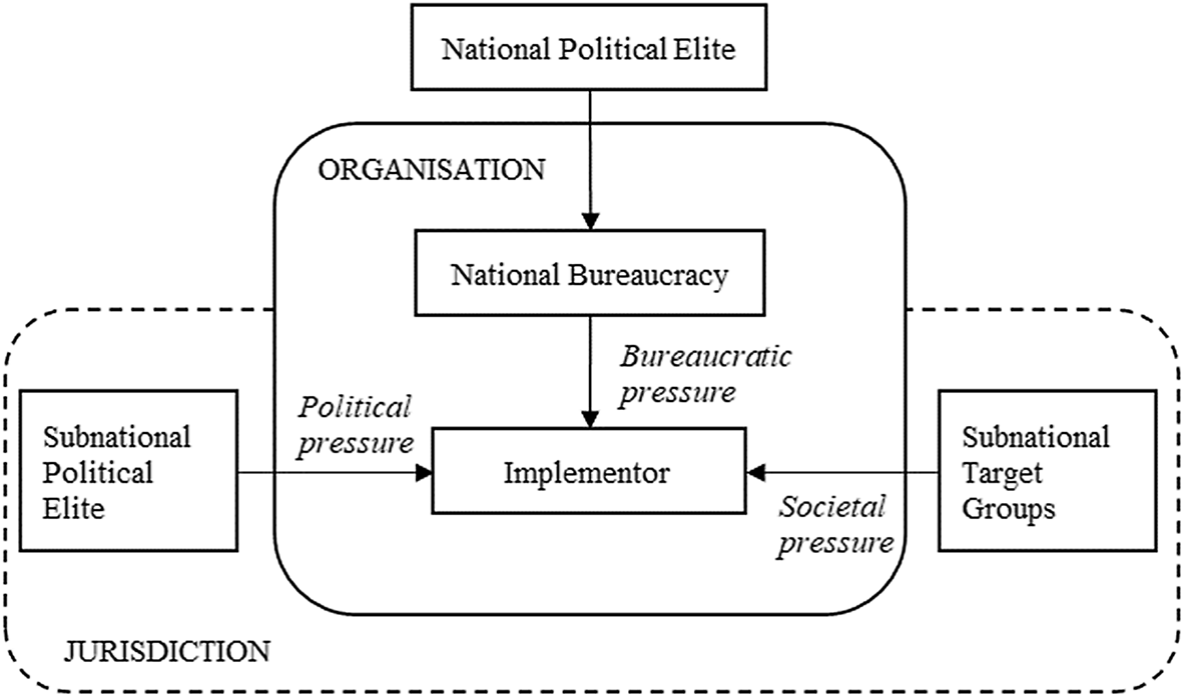
Policy implementation at the subnational level. *Source*: adaptation from Grindle (1980: 205)

With regard to subnational politics, an important role in the achievements of national policy and programme objectives is played by the organisation responsible for policy execution, or *implementor* (Grindle, 1980), acting in a specific area (e.g. a region, a province or a municipality). In its effort to achieve central policy objectives without causing social conflicts, the implementor works under the burden of several (often conflicting) pressures that are bureaucratic, political and societal. First, the implementor is accountable to its national bureaucracy (along a vertical hierarchical line) for achieving the goals specified by national legislation and programmes. Second, it responds to the political leaders elected in its territory (region, province or municipality). Third, it is constrained in its work by (both the expectations and interests of) the target groups of a policy or programme, namely the territorial economic elites. Under these pressures, the content of national policies and programmes may change significantly during implementation, while the public policy (or programme) moves from the national to the subnational level (Fig. 2).

Capacity is the ability to manage the physical and immaterial resources required to deliver public policies and governmental programmes: funds, personnel, talent, appropriations, equipment, knowledge and information, leadership, energy and time (Painter & Pierre, 2005). Considerations on subnational capacity need to be contextualised to the specific case of overseas Europe. Because of their geographical remoteness from the European continent and its major (political) centres, EU’s ORs and OCTs can be compared to the rural and urban peripheries of mainland Europe (Interview 2). Studies on local economic development have stressed the disadvantages of peripheral areas. These disadvantages include the costs linked to their remoteness (i.e. the increased travel costs due to longer distances), the sparsity of population (that prevents, for instance, economies of scale and critical mass for knowledge production) and a weak influence on central governance (because of their distance from the national capitals) (Copus, 2001).

## Methodology

The final purpose of research is producing general knowledge (King et al., 1994). Even when (qualitative) research starts from one case, these single-case studies allow a certain degree of inference from the qualities specific to the case studied to a more general explanation (Gerring, 2004). The generalisation to cases not studied always entails some risk of mistaken inference. However, “extremes of a complete inability to generalize from a case and a warrant for broad generalizations from a single case are relatively infrequent” (George & Bennett, 2005: 124). In particular, when single cases are not purely inductive exercise but are guided by theoretical reflections (i.e. theory-driven case studies) embodied in an analytical framework (Sect. 2), the generalisability of their findings is enhanced (Yin, 2003). Reliance on an analytical framework links the insights from one case to earlier analytic work on a higher number of previous cases and observations on which the analytical framework is built (Rueschemeyer, 2003).

To sum up, the use of an analytical framework makes theory-driven single-case studies strong enough to offer persuasive causal explanations. The underlying processes analysed in the case of Reunion and the conclusions that will be developed can suggest explanations common to other (remote) regions of France (and Europe) in their effort of implementation of biodiversity policies. In order to understand the process of implementation of national biodiversity policy at regional level in Reunion, data were collected through document analysis and interviewing.

Documents included primary sources such as official documents (e.g., laws, strategies and other policy documents) issued by both the national government of France and subnational authorities in Reunion. Few secondary sources were also consulted both from academic and grey literature (e.g. reports from international organisations). The study relied on internet sources for most recent updates about the organisation of the French state and regions, or current policy developments.

Primary sources of data also included semi-structured interviews. A total of eight participants were interviewed from international, national and regional organisations. The number of interviewees has included both employees of public agencies and personnel of non-governmental organisations active in the field. Due to the COVID-19 pandemic, interviews in the field took a full semester^5^ (from March to November 2021); some interviews were conducted online. Content analysis was carried out on interviewers’ notes and combined with interviews’ transcripts generated automatically for online interviews. Interviewees’ answers were encoded, systematised, cross-checked and connected to present a valid and comprehensive picture of the findings (Sect. 4). As argued by Mosley (2013: 11), “converting interview transcripts and answers into more discrete concepts and categories always involves some type of interpretive work”. However, the fact that this work is the product of the punctual analysis of the answers by at least two interviewers ensures a good degree of objectivity. Finally, to ensure anonymity of the data sources, interviews have been numbered (from 1 to 8).

## Biodiversity policy in France and Reunion

After four decades of institutional stasis, biodiversity policy in France experienced a major change in 2016 when the country adopted *Loi n°2016–1087 pour la reconquête de la biodiversité, de la nature et des paysages* (Table 1) (OECD 2016b; Interview 6). In fact, already in the early 2000s, some changes took place in France’s environmental policy with the adoption of Grenelle Law 1 (2009) and Grenelle Law 2 (2010) that have institutionalised public participation in environmental matters. The new Biodiversity Law of 2016 revised the existing legal framework for biodiversity protection and established the *Agence Française de la Biodiversité* with the mission of coordinating the various public agencies responsible for biodiversity. Created in 2017, this national biodiversity agency was later replaced by the French Office for Biodiversity^6^ (*Office français de la biodiversité*) (OFB) in 2021 (Interview 3).

**Table 1:** Biodiversity policy in France and Reunion

Type of document	France	Reunion	Related international and EU commitments
Legislation	*Loi pour la reconquête de la biodiversité, de la nature et des paysages* (2016)		Convention on Biological Diversity (1992)
Strategy	Second National Biodiversity Strategy (2011)	*Stratégie Réunionnaise pour la Biodiversité* (2014) *Stratégie de conservation de la flore et des habitats de La Réunion* *Stratégie de lutte contre les espèces invasives à La Réunion*	Convention on Biological Diversity (1992) Aichi Targets (2010) EU Biodiversity Strategy (2010, 2020)
	*Stratégie nationale pour la mer et le littoral* (2017)	*Document stratégique de bassin maritime Sud océan indien* (2020–2026)	Marine Strategy Framework Directive (2008) Marine Spatial Planning Directive (2014)

France has also issued two national strategies for biodiversity. They have complied with the Convention on Biological Diversity (CBD) that the country ratified in 1994^7^ (first National Biodiversity Strategy, 2004), and the Aichi Targets and the EU Biodiversity Strategy issued in 2010 (second National Biodiversity Strategy, 2011) (MEDDTL, 2011). Through its first National Biodiversity Strategy, ‘France has, for the first time, integrated the challenges of overseas entities into a national policy of biodiversity conservation, with an action plan specifically dedicated to these entities’ (Benzaken & Renard, 2011: 25). The second National Biodiversity Strategy confirms the country’s commitment to the conservation of biodiversity on both mainland France and in its overseas territory with an encouragement for the adoption of regional (and local) strategies for biodiversity conservation through a participatory approach (MEDDTL, 2011). The document also stresses the importance of both protection and restoration of biodiversity and confirms the relevance of protected areas as a tool for nature conservation (MEDDTL, 2011). A new (post-2020) National Biodiversity Strategy is being developed by the MET at the time of writing (Spring 2022) (Interview 3).

France has been compliant also with other international and EU commitments relevant for biodiversity. For instance, the country adopted the *Stratégie nationale pour la mer et le littoral* in 2017 in compliance with the Marine Strategy Framework Directive (MSFD) and the Marine Spatial Planning (MSP) Directive.

### Implementing biodiversity policy in France

**Fig. 3:**
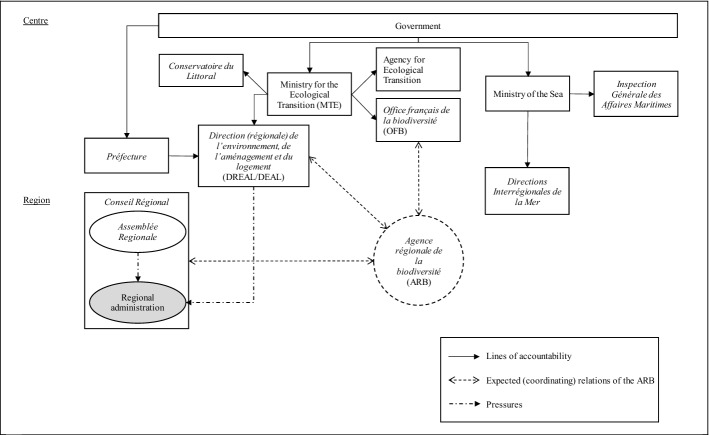
Implementing biodiversity policy in France

In France, environmental policy is co-ordinated at the central level by the Ministry for the Ecological Transition^8^ (*Ministère de la transition écologique*) (MET) and conducted at the subnational level (in regions and *départements*) by decentralised regulatory bodies.^9^ These bodies are the *Direction régionale de l’environnement, de l’aménagement et du logement* (DREAL) in the regions—called *Direction de l’environnement, de l’aménagement et du logement* (DEAL) in overseas France—and the *Directions départementales des territoires* and *Directions départementales des territoires et de la mer* in the *départements*^10^ (Fig. 3). The work of MET is supported by two implementing agencies, i.e. the Agency for Ecological Transition (*Agence de la Transition Écologique*) and the OFB. The Coastal Conservatory (*Conservatoire du Littoral*) (CDL) has a special role in the protection of the coast through acquisition and restoration of threatened coastal areas (Interview 6).

The Ministry of the Sea (*Ministère de la Mer*) focuses on policy initiatives for the sea, and the General Inspectorate of Maritime Affairs (*Inspection Générale des Affaires Maritimes*) carries out inspections. The Ministry also has decentralised services responsible for implementing maritime policies in each basin, i.e. the Interregional Directorates of the Sea (*Directions Interrégionales de la Mer*).

While environment and biodiversity in France are ruled by national laws, it is the decentralised administration—made up of regions, *départements* and municipalities—that is responsible for policy implementation in these areas. The French “region”, managed by the Regional Council (*Conseil Régional*), constitutes the highest level of subnational governance, although there is no hierarchy among subnational authorities (i.e. regions, *départements* and municipalities). The Regional Council works as a strategic coordinator producing regional schemes and strategic documents with competences mainly on economic development and biodiversity. Indeed, regions in France have no legislative power with very few exemptions (e.g. for subventions and funding) (Interview 1).

In the Regional Council, the administrative personnel (i.e. the civil servants) is accountable to the (political) Regional Assembly of elected officials (*Assemblée Régionale*). The Regional Assembly has decision-making responsibilities, decides on the distribution of the budget among the items on the agenda (e.g., infrastructures, transport and environment) and guides the activity of the regional administration that functions as the *implementor*.

France has embarked on a decentralisation process since the early 1980s. The distribution of powers in the French territorial organisation has been changed, clarified and improved under several laws in the last decades. In this effort of reorganisation, the NOTRe Act^11^ (2015) represents a milestone (OECD 2016a; b). As a result of these institutional changes, regions in France have taken over a leading role in important policy domains such as biodiversity, climate change and several others (e.g., infrastructures, transport and environment) (Interview 3). In the area of biodiversity, important powers are nonetheless shared by the regions with their *départements*; the latter is competent, for instance, for the management of natural areas and water use. Below the *départements*, the municipalities prepare local urban planning documents, collect and process household waste and manage aquatic environments among other tasks (OECD 2016a).

Decentralisation in the area of biodiversity has had three major consequences. First, the central government is still very present in the regions through its decentralised services (e.g. DREAL/DEAL) that respond to regional prefects. However, it has lost part of its co-ordinating capacity due to less legal power and fewer financial resources allocated for this role. This has weakened the central government’s ability to guide local policy-makers and local economic actors for a coherent application of national strategies. Second, the powers of subnational elected representatives have increased—particularly during policy execution—and led to many cases of uneven implementation of national policies that become dependent on the will of local politicians. This can jeopardise policy implementation to the detriment of biodiversity. Third, decentralisation has led to a plethora of competent bodies; responsibilities often overlap between the central government, its national decentralised services and national public agencies, on the one hand, and multiple layers of subnational administration authorities, on the other. In the presence of a weak centre-local coordination, such overlaps in competences are likely to hinder the implementation of national policies, particularly in area of environment (OECD 2016a; 2016b).

### The case of Reunion

Reunion is both a region and a *Département d'Outre-mer* (DOM) of France; it is also an OR of the EU (Tanguy et al., 2017). Environmental governance in Reunion is multi-layered: it includes national, regional, departmental and community-level authorities. The national government is present in the island through the *Préfecture* that supervises the activities of the decentralised services of the state such as the DEAL. Established in 2011, the DEAL executes and enforces at the subnational level the MET’s decisions about biodiversity. The Regional Assembly includes 45 elected representatives who guide the 2,300 civil servants working in the regional administration (Interview 7).

As a region of France, Reunion is bound to comply with several international conventions signed by the State such as the CBD (Tanguy et al., 2017). In its action, the Region is also guided and constrained by the national legislative framework (Table 1) and its alignment with EU law. The adoption of a national Biodiversity Law in 2016 (see above) has provided the means for a stronger action of the Region in the area of biodiversity through better organisational coordination and longer strategic planning at regional level (Interviews 5, 6 and 7).

First, the Biodiversity Law gives the French regions the possibility to create a regional biodiversity agency (*Agence régionale de la biodiversité*) (ARB) (Interview 3) conceived as the regional translation of the OFB (Interview 3 and 7). Although this is not an obligation imposed from the central government, Reunion has committed to the creation of its own ARB based on a partnership between the State (through the DEAL), the Regional Council and the OFB. The ARB is expected to start its activity in 2022 (Interview 3). The mission and lines of action of the new agency are currently being developed in consultation with stakeholders.^12^ Among its different lines of action, the ARB will be in charge of EU funding (that is crucial for the island’s subnational capacity) and regional cooperation across the entire Indian Ocean (Interviews 3 and 7).

Second, Reunion has issued regional strategic documents. In compliance with the Biodiversity Law 2016, Reunion has adopted the *Stratégie Réunionnaise pour la Biodiversité* (SRB). The document translates the second National Biodiversity Strategy of 2011 at the regional level and constitutes the first regional strategic document on biodiversity bringing together State, Region and *Département*. The SRB has been complemented by two other strategic documents: *Stratégie de conservation de la flore et des habitats de La Réunion* and *Stratégie de lutte contre les espèces invasives à La Réunion*. Another regional strategy^13^ was recently adopted for the period 2020–2026^14^ as subnational implementation of the *Stratégie nationale pour la mer et le littoral* of 2017 (Tanguy et al., 2017; Interview 3).

The current SRB will be followed by a new regional biodiversity strategy that is currently being formulated with the engagement of different stakeholders (Interviews 7 and 8). The new SRB will be shaped around the lines of action of the ARB to ensure more coherence within the Region (Interview 3). However, some interviewees shared doubts about the effective alignments of the ARB’s future action with local concerns and priorities; frictions between regional and local authorities have been reported during interviews in Reunion (Interviews 5 and 6).

### Policy results: protection of areas, habitats and species

Biodiversity policy in France relies on a set of policy instruments that include regulatory tools (e.g. protection of areas, habitats and species), economic measures (i.e. those financing actions against biodiversity loss) and other interventions (from information campaigns to restoration initiatives through direct intervention) (OECD 2016a). The section will mainly analyse the regulatory instruments put in place in Reunion for biodiversity protection.

The momentum given to protected areas by the second National Biodiversity Strategy (Sect. 4.1) has helped the country reach the international objectives of protecting at least 17% of its land area and 10% of the waters under its jurisdiction in line with the CBD and the Aichi targets (OECD 2016b). In fact, one third of French waters (both in metropolitan and overseas France) are now marine protected areas (Claudet et al., 2021). France has also established protected areas as part of the Natura 2000 network. This network gives effect to the two EU Nature Directives through the creation of special conservation zones for habitat types (under the Habitats Directive) and special protection zones for wild bird species (under the Birds Directive) (OECD 2016a). However, the Nature Directives do not apply to Reunion and all other DOM-ROMs (Interviews 3, 6 and 7).

The level of protection of areas is higher in overseas France according to the OECD (2016a). In particular, Reunion has put in place a good regulatory framework for the protection of its areas both on land and in the seas (Interview 5). Protection measures seem to be even stronger for marine than terrestrial areas (Interview 4). Although the Regional Council can establish regional parks and reserves, all protected areas of Reunion have been established by the State (Interviews 7 and 8). The *Parc national de La Réunion* was created in 2007 and covers 42% of the territory of the island; it is fully funded by the State (through the MET). After some initial issues, a management plan was adopted in 2014, i.e. *Charte du parc national*. The island also has two national natural reserves. The first is the *Réserve nationale marine de La Réunion* (created in 2007); it has its own management plan that is elaborated in collaboration with relevant stakeholders and funded by both the State and the Region. The second is the *Réserve de l’étang de Saint-Paul* (created in 2008) (Tanguy et al., 2017; Interviews 3 and 6). The Region (as well as the State and the *Département*) is present in the management bodies of both the National Park and the Marine Reserve (Interview 3). Several other areas are under some form or protection (as reserves) for habitats and species (Tanguy et al., 2017).

France has improved its protection of certain habitats; it has also elaborated action plans to restore and conserve populations of endangered species that have proven to be successful (OECD 2016a). National action plans for species conservation are being implemented in Reunion among other ORs of France (Benzaken & Renard, 2011). Finally, France has formulated strategies and action plans to combat invasive alien species (IAS) (Benzaken & Renard, 2011; OECD 2016a). Species and habitats are protected in Reunion under several regional strategies (Sect. 4.2). IAS represent a major threat to the local flora that is still in danger in Reunion despite the numerous policy initiatives (Interviews 3, 5 and 7).

## Major constraints in implementation

Many factors located at the subnational level of governance can intervene during implementation and ease or hinder the achievement of stated policy objectives. According to policy studies, subnational politics and subnational capacity are crucial in this process. This section discusses the major findings related to the case of Reunion along these two dimensions.

### Subnational politics and a multitude of pressures

The interactions among a wide range of actors and organisations influence the implementation of any national policy and programme. In its path from the national to the subnational level, the content of national policies and programmes can be significantly diverted from its original design. In the French institutional setting, the national biodiversity policy issued at the central level is implemented by the decentralised administration of the country. This article has focussed on the region and, more precisely, the regional administration of Reunion in its role as *implementor*. In general, the implementor works under the pressures of the central bureaucracy, the political leaders elected in its territory—a region in this case—and the target groups and economic elites present in the region (Fig. 2). In the case of Reunion, the regional administration of the island is under bureaucratic, political and societal pressures from the national administration (through the DEAL), the regional elected bodies (i.e. Regional Assembly) and vested socio-economic interests (mainly from fisheries, tourism and land use for urban development).

First, although the process of decentralisation in France has reduced the power of the central government, its guidance during policy implementation is still quite strong and embodied by the DEAL. In fact, this has benefited biodiversity protection in Reunion; for instance, the protected areas of the island have been established by the State (Interviews 7 and 8). Despite the increasing power attributed to the Regional Council in the last decades, the regional administration is still a new actor in environmental policy. The formal recognition of responsibilities in the environmental domain has not been followed by a stronger action of the regional administration in this field. In addition to the bureaucratic pressures exerted “from above” by the central administration, Reunion’s public administration has been exposed to a bureaucratic pressure coming “from below”. The *Département* is the island's largest landowner with more than 100,000 ha of land (Tanguy et al., 2017). This administrative layer has traditionally been directly involved (and adequately staffed) in the environmental domain often as a partner of the central administration in the execution of national policy initiatives (Interviews 6, 7 and 8). However, this may create tensions with the region and clashes of competences now that its powers have been increased. Ambiguities in the attribution of responsibilities constitute a recurrent externality of the decentralisation undertaken in France (OECD 2016a; b).

The result is that biodiversity governance in Reunion is affected by bureaucratic pressures (from above and below) on the regional administration and frictions across an excess of competent and uncoordinated administrative agencies. This determines conflictual power relationships in the Region that impact negatively on policy implementation (Interview 3). The major national reforms, i.e. the adoption of the Biodiversity Law of 2016 and the creation of the OFB, have improved coordination and planning but not sufficiently. Problems during policy implementation have also affected marine spatial planning. France issued its national strategy for MSP for the whole country, including its ORs, in 2017 (Table 1), but the blurred demarcation of competences has jeopardised its execution (Interview 1).

Second, the regional administration executes its functions of implementor under the political pressure of regional politicians. Decentralisation in France has empowered subnational elected representatives with more autonomy, particularly during policy implementation. In this context, the implementation of national laws, strategies and programmes largely relies on the political will of regional, as well as local, policy-makers to execute national policy priorities. The delivery of national policies such as the ones governing biodiversity has become dependent on the action of these politicians.

In Reunion, a strong political attention has been diverted to biodiversity in the last years, but mainly for economic reasons and as long as conservation serves economic development (Interviews 6 and 7). In the island, the good conservation of the environment becomes important only if it is beneficial for the economy of the region (Interview 1). The priority on the regional political agenda in Reunion remains economic growth and job creation; unemployment is indeed quite high and an alarming phenomenon among younger people (IEDOM, 2015). The rate of unemployment was 48% in 2020; it was 26% for people among 15 and 29 years of age in the same year.^15^

Third, biodiversity in Reunion is mainly threatened by natural phenomena (e.g. coastal erosion from wind and sea waves made more severe by climate change), pollution (from the land-based sources) and human activities (Interview 1). The implementation of policy interventions for the protection of biodiversity on the island is often under the pressure of many conflicting uses of the marine and coastal environment by strong socio-economic interests. Elected officials are reluctant to adhere to new national and international obligations for the protection of biodiversity whenever it can contrast with the electorate’s demands and the preferences of strong lobbies in sectors like fisheries, agriculture and tourism (Interviews 4 and 6).

### Subnational capacity and limited resources

The capacity of the European ORs is usually limited by their remoteness, insularity and small dimension. These factors may determine weaker economies with an impact on the availability of resources for policy implementation.

In Reunion, public funding for the environment is still very limited due to other socio-economic priorities that crowd the political agenda. The prioritisation of economic growth and job creation influences the allocation of funds (Tanguy et al., 2017; Interviews 4 and 6). In addition, a large part of the funding allocated to the environmental area is largely used for water management. In other words, biodiversity protection is neglected in terms of budget allocation within the same environmental policy area. To some extent, EU funding has compensated the lack of adequate national and regional funds; yet multiple short-term projects (funded by the EU) are necessary to finance long-term interventions for biodiversity (Interview 5).

Even when financial resources are available, the island may lack dedicated personnel because of the size of its population, which can play in favour of inaction. For instance, once France had issued its national strategy for MSP (2017), many ORs lacked capacity (in terms of human resources and technical skills) to implement this strategy and produce their own marine spatial plan (Interview 1 and 5).

## Conclusion

As biodiversity hotspots, the ORs of Europe can play an important role in the conservation of nature both on land and in the sea. The protection of the coastal and marine environment is particularly important due to the services provided by the oceans and their ecosystem to humans. Furthermore, regions are essential for biodiversity protection because of the role they play in the subnational implementation of national and international environmental policies and political strategies. By focussing on the case of Reunion, the article has unravelled how politics and capacity at the subnational level impact the achievement of national objectives for biodiversity as well as France’s commitment to international and EU targets. Subnational politics is the result of bureaucratic, political and societal pressures that intervene in the process of implementation. They can change, divert and water down policy intents, while national policies and programmes move from the national to the subnational level of governance. Matters of subnational capacity linked to the scarce availability of resources add further constraints to effective implementation.

The case of biodiversity policy in Reunion confirms obstacles that seem to be common to other French ORs. According to the OECD (2016a), implementation of environmental policy in France’s ORs can be delayed by the same devolution of powers that the country has promoted in the last decades. Indeed, this has generated overlaps of competences that make policy implementation conflictual in the absence of smooth coordination mechanisms among public agencies, and between bureaucratic and political lines of accountability. Failures in policy implementation in overseas France are also due to the weak awareness and mobilisation of local stakeholders in biodiversity decision-making. Finally, weak technical capacity at the regional level can hinder the execution of both international and national policies (OECD 2016a).

The in-depth analysis conducted for Reunion and its nature of theory-driven case study has produced possible causal explanations for a better understanding of subnational implementation that can be valid also for other European ORs. The limitations on generalisations typical of (qualitative) research on single cases (Sect. 3) call for future, possibly comparative, research among ORs of the same countries and of different Member States of the EU. For the same limitations, the findings cannot claim a complete generalisation to all overseas entities of France and Europe characterised by very different institutional arrangements and socio-political contexts. However, these findings raise an important call for action from the public actors of mainland France and other European countries with ORs (e.g. Portugal and Spain) along a few pathways of improvement. Table 2 summarises these pathways.

**Table 2:** Pathways of improvement

1	Improve the national-subnational linkage through involvement and coordination
2	Reinforce horizontal coordination through a regional strategy
3	Strengthen public engagement
4	Enhance capacity for biodiversity protection through transnational collaboration

First, in the complexity of joint action that characterises the French state, the response of subnational agencies to (central) policy mandates and the coordination among them is a pre-requisite for avoiding implementation failures. The involvement of subnational authorities from overseas seems to be rather weak in the adoption of national strategies. According to the OECD (2016a), under the influence of the Grenelle laws, the process of adoption of the second National Biodiversity Strategy was more participative than the one leading to the previous strategy. However, the involvement of (overseas) subnational authorities was still rather weak. Better coordination should also be pursued between national and regional competent authorities. Political and administrative efforts are somehow dispersed in the absence of efficient coordination among regional actors and between regional and national competent authorities. The creation of a regional biodiversity agency in Reunion (i.e. ARB) could improve this aspect (Interview 5).

Second, horizontal integration as well as vertical integration is needed. Indeed, one aspect that was stressed during the field research conducted in Reunion is the lack or weakness of policy coherence. The conservation of biodiversity is a cross-cutting issue that is difficult to put into practice because of the strong interactions with several sectors: agriculture, urbanism, etc. (Interview 5). In line with the second National Biodiversity Strategy, Reunion has adopted a regional strategy for biodiversity that is expected to help integrating biodiversity conservation into other policy areas and across the several levels of authority (i.e. State, Region and *Département*). The engagement of a broad range of territorial stakeholders in the preparation of a new regional strategy may bring this effort of coherence even further.

Third, theoretical insights from the study of policy implementation have for long time stressed the crucial role of the policy receivers (or target groups) for the effectiveness of any policy initiative. Participatory mechanisms are recommended for the purpose of solving conflicts of use (Sect. 2). Adequate communication with stakeholders and awareness building needs to be enhanced in Reunion (Interview 1). A culture of public participation is still too weak in the island on two sides. On one side, decision-makers are not used to involve citizens in public decision despite some rhetorical commitment. On the other side, citizens are not familiar with public engagement (Interview 5). Public participation in the Region is slowly developing, but ultimately decisions are still taken behind closed doors. Citizens’ pressure on decision-makers is traditionally very weak and punctuated by episodes of civil disobedience, protest and demonstrations. On some occasions, citizens’ disagreement can find expression in violent form of opposition (e.g. death threats) (Interview 4). Beyond Reunion, the OECD (2016b) stressed that public engagement in France needs to be strengthened across all policy sectors including the environmental area.

Fourth, while the concurrent action of the State and the Region has led to concrete improvements in the conditions of protected areas, the regional strategies for the protection of species have achieved weaker results, especially for the flora (Sect. 4.3). Stronger protection interventions do not only need more harmonious legislative and regulatory frameworks (that Reunion has been able to put in place), and coordination across management bodies. They also depend on available knowledge on the status of habitats and species in order to correctly inform decision-making. However, the production of more knowledge and its delivery to decision-makers as science advice remains challenging in Reunion because of the internal capacity of the island. Therefore, France should not only improve coordination between the central administrative bodies and the related public agencies with its overseas entities, but also promote the collaboration of its DOM-ROMs with the overseas entities of other Member States of the EU so to strengthen capacity through the exchange of knowledge and mutual learning (Tanguy et al., 2017).

## Footnotes

1 The ORs include: the Canary Islands (Spain); Azores and Madeira (Portugal); and the French ORs (Fig. 1).

2 Only two of these overseas entities are not islands: *Terre Adélie* and French Guiana.

3 Source: https://www.insee.fr/fr/statistiques?theme=0 (last access: 22.04.2022).

4 The BEST Initiative seeks to promote biodiversity conservation in the ORs and OCTs of the EU (source: https://ec.europa.eu/environment/nature/biodiversity/best/about/index_en.htm; last access: 05.05.2022). IFECOR is a French initiative for the protection of coral reefs (source: https://ifrecor.fr; last access: 09.05.2022).

5 We are thankful to Rachel Bitoun (*Institut de Recherche pour le Développement*, France) for her help during data collection.

6 Source: https://ofb.gouv.fr (last access: 18.03.2021).

7 France is Party to the CBD also on behalf of its overseas entities. However, the implementation of policy actions for biodiversity conservation in the overseas entities differs according to the legal status of each entity. To simplify a complex matter, we can distinguish two major situations (with internal variations and exemptions): the French state is the main authority for biodiversity conservation in the *départements et regions d’outre-mer* (DOM-ROM) like in the case of Reunion, while biodiversity conservation (and more broadly environmental matters) fully falls under the territorial jurisdiction of the local authorities in the OCTs (Benzaken & Renard 2011) (Fig. 1).

8 Source: https://www.ecologie.gouv.fr (last access: 18.03.2021).

9 Source: https://www.ecologie.gouv.fr/services-deconcentres-du-ministere (last access: 18.03.2021).

10 Source: https://www.ecologie.gouv.fr/services-deconcentres-du-ministere (last access: 07.01.2022).

11 Full name: *Loi portant sur la Nouvelle Organisation Territoriale de la République* (NOTRe).

12 The Grenelle laws (see above) have largely influenced the development of the societal engagement in public decisions in Reunion. In addition, the movement of the *Gilets Jaunes* has played a role in opening up public decision-making to societal representation in France. However, this participatory dimension is quite new in Reunion and still in a phase of development (Interview 3).

13 *Stratégie pour la mer et le littoral du bassin maritime Sud Océan Indien (La Réunion – Mayotte – Terres Australes et Antarctiques Françaises)* (source: http://www.reunion.gouv.fr/consultation-du-public-sur-la-strategie-pour-la-a6997.html; last access: 07.10.2021).

14 Source: http://www.dm.sud-ocean-indien.developpement-durable.gouv.fr/le-document-strategique-de-bassin-maritime-dsbm-r247.html (last access: 21.10.2021).

15 Source: https://www.insee.fr/fr/statistiques/5354321#consulter (last access: 27.04.2022).
